# Integrating FT-ICR MS and Machine Learning to Forecast Acid Content Across Boiling Cuts

**DOI:** 10.1021/acs.analchem.4c04522

**Published:** 2025-02-26

**Authors:** Jussara V. Roque, Wilson J. Cardoso, Deborah V. A. de Aguiar, Gabriel F. dos Santos, Alexandre de O. Gomes, Iris Medeiros Júnior, Gesiane da S. Lima, Boniek Gontijo

**Affiliations:** † Laboratory of Chromatography and Mass Spectrometry, Institute of Chemistry, Federal University of Goiás, 74690-900 Goiânia, GO, Brazil; ‡ CENPES, PETROBRAS, 21941-915 Rio de Janeiro, RJ, Brazil

## Abstract

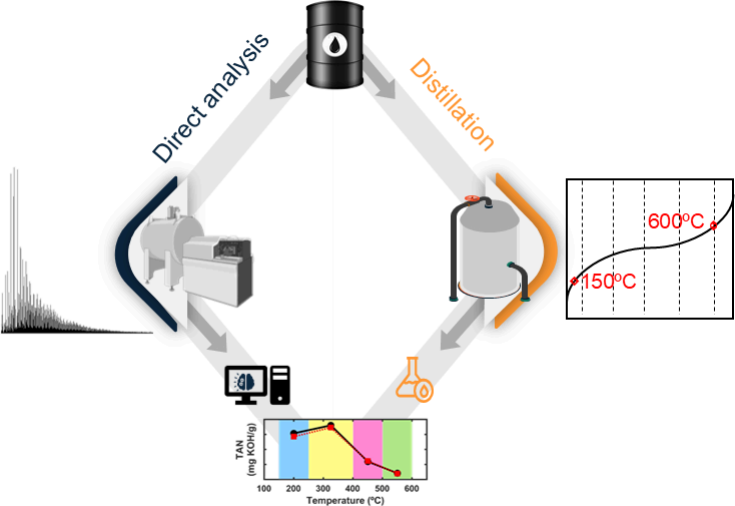

In this study, we introduce a pioneering approach that leverages advanced machine learning and ultrahigh-resolution Fourier transform ion cyclotron mass spectrometry (FT-ICR MS) data to predict the distribution of the total acid number (TAN) in true boiling point (TBP) distillation cuts from crude oil. By employing partial least-squares (PLS) regression and ordered predictor selection (OPS), we achieved robust predictive models with high accuracy, evidenced by low root-mean-square error of calibration (RMSEC) and strong correlation coefficients (Rc). Our analysis of 36 diverse crude oil samples revealed significant variations in chemical composition, with nitrogen- and oxygen-containing compounds playing key roles in influencing TAN values. Through the use of volcano plots, we identified critical molecular classes that drive changes in TAN. The predictive models demonstrated remarkable consistency between predicted and actual TAN values, particularly in samples with a higher TAN, further validating their reliability. Significantly, our method overcomes the limitations of traditional ASTM testing by requiring smaller sample volumes while still providing accurate TAN predictions. This novel approach offers a powerful new tool for the molecular characterization and behavioral forecasting of complex mixtures, enabling a more efficient pathway for sample analysis when resources are limited.

## Introduction

Petroleum is a complex mixture of hydrocarbons that includes a polar fraction (NSO-based compounds), which plays a significant role in determining crude oil quality, influencing its economic value and refining potential.^1−5^ In this context, the application of advanced technologies capable of accurately characterizing the chemical composition of crude oil has been crucial, enabling correlations with its physicochemical properties to be established. The total acid content in crude oil is a key physicochemical property that directly affects its market value and processing. It is closely linked to corrosion risks in refineries and significantly impacts the oil’s stability and quality. This property is not only attributed to carboxylic acids but also to all organic or inorganic compounds able to react with a base species, such as phenolic compounds, carbazole, amines, thiophenols, mercaptans, esters, salts of heavy metals, and hydrolyzed salts.^3,6,7^ The crude oil acid levels are frequently assessed in terms of total acid number (TAN) measurement, defined as the amount of KOH (mg) necessary to neutralize the acid compounds in 1 g of crude oil.^3,4,6,8,9^ Despite the petroleum industry having established laboratory protocols for determining TAN, these routine analyses are time-consuming, involving distinct experiments, equipment, and a significant sample volume.^3,10^ TAN measurements typically need significant amounts of crude oil and cannot be conducted on small samples or when the crude oil has a high water content. Additionally, to understand the acidity comprehensively, it is often necessary to distill the crude oil into various fractions and determine the TAN for each distillation cut. This process is crucial but further complicates the procedure, especially when the amount of crude oil is limited, making it a bottleneck.^6,9,11^

Ultrahigh resolution mass spectrometry techniques, such as Fourier Transform Ion Cyclotron Mass Spectrometry (FT-ICR MS), combined with multivariate analyses, have yielded substantial insights into the composition and properties of petroleum.^5−8,12−16^ The FT-ICR mass spectrum can contain up to ∼50,000 peaks. In some cases, the standard data processing methods are often not sufficient to visualize all generated data simultaneously and thoroughly explore the resulting analyses.^17,18^ Hence, it is recommended to employ advanced approaches such as machine learning to assess the data. Machine learning methods have been successfully applied to predict properties and have proven their effectiveness in evaluating crude oil properties.^4,6,12−16,19^

Approaches combining FT-ICR MS and chemometric tools have been revealed as an effective strategy to manage these challenges. Vaz et al. developed uni- and multivariate calibration methods combining O_2_ compounds data accessed by ESI (−) FT-ICR MS and applying chemometric tools to predict the TAN of crude oils.^6^ Terra et al. used ESI (−) FT-ICR MS combined with PLS regression and variable selection methods to estimate the TAN of Brazilian crude oil samples.^7^ Afterward, Terra et al. applied ESI (−) FT-ICR MS coupled with chemometric tools to predict the TAN of distillation cuts.^20^ Although these studies applied FT-ICR MS data combined with chemometric tools, they presented some limitations: most of them were developed to predict the TAN only in crude oils,^6,7^ considering only the data of O_2_ class,^6^ or developed to predicted the property in distillation cuts individually.^20^ In contrast, we have leveraged multivariate calibration models using FT-ICR MS data and chemometric tools capable of accurately predicting the TAN (in a diverse range) of Brazilian crude oils and their distillation fractions without requiring distillation. The combination of FT-ICR MS and chemometric methods has been proven to have great potential to evaluate other petroleum properties.^6,13−16,19^ To continue addressing significant issues in the petroleum industry, the same approaches applied here could be extended to predict other relevant petroleum properties and attempt to address asphaltene deposition based on FT-ICR MS data and gas-phase fragmentation, which is one of the most significant bottlenecks in the petroleum sector.^21−24^

Particularly, PLS regression is advantageous for FT-ICR MS data due to its ability to handle complex and high-dimensional data sets.^25^ PLS is effective in modeling relationships between large sets of predictors and responses, making it suitable for FT-ICR MS data, where the number of variables often exceeds the number of samples. This method reduces dimensionality while preserving essential information, facilitating the identification of significant variables correlated with specific outcomes.^26,27^ Ordered Predictor Selection (OPS) is a variable selection method that enhances the interpretability and predictive performance of multivariate models based on PLS. OPS works by systematically ranking variables based on their contribution to the predictive power of the model. This ranking allows for the identification of the most relevant predictors while minimizing the influence of noise and irrelevant variables.^28,29^ Such methods offer a significant advantage, as they can understand complex patterns without requiring prior knowledge of the relationship between independent and dependent variables.^16,29−32^ Together, these methods address the challenges of multicollinearity and overfitting/underfitting inherent in high-dimensional mass spectrometry data, leading to more robust and accurate analytical models.

At the core of this research is the development and validation of predictive models for determining the TAN values of crude oils and their distillation cuts. Specifically, the study focuses on building and evaluating multivariate calibration models capable of accurately predicting the TAN of Brazilian crude oils and their distillation fractions. By applying machine learning to FT-ICR MS data, these models allow for both direct and indirect determination of TAN values, streamlining the analysis of complex petroleum samples. The efficacy of these predictive models lies in their ability to serve as reliable alternatives to traditional laboratory methods,^33,34^ significantly reducing the time and carbon emissions with TAN determination. In scenarios in which conventional methods are impractical due to sample scarcity, this predictive model provides an efficient solution for characterizing crude oils, eliminating the need for extensive distillation and titration procedures.

## Experimental Section

### Materials

HPLC-grade toluene was purchased from Tedia Company (Fairfield, USA). Sodium trifluoroacetate (NaTFA), HPLC-grade methanol, and ammonium hydroxide (NH_4_OH) were purchased from Sigma-Aldrich (St. Louis, USA). Thirty-six crude oil samples were provided by the Centre of Research, Development, and Innovation Leopoldo Américo Miguez de Mello (CENPES, Petrobras, Rio de Janeiro, Brazil).

### Standard Method of TAN Determination

All crude oil samples were distilled into fractions (these experiments were conducted at CENPES), and the TAN values of both the crude oils and the fractions were determined by the reference method at CENPES according to the ASTM D664-09 procedure^34^ using potentiometric titration. It is important to highlight that only crude oil samples were used in the development of this work. The distillation fractions were used solely to obtain the reference TAN values.

### Sample Preparation and Mass Spectrometry Analysis

Crude oils were dissolved in toluene:methanol (50:50, *v/v*) at a concentration of 0.500 mg mL^–1^. To each sample was added 50 μL of NH_4_OH to improve ionization. A total of 5.0 μL of a NaTFA methanol solution (1.0 mg mL^–1^) was added to each sample and used as an internal standard.

Mass spectrometry analyses were conducted using an FT-ICR MS 7T SolariX 2xR instrument (Bruker Daltonics, Bremen, Germany) coupled with an ESI source in negative-ion mode. Negative ESI was chosen to enhance the ionization of species with acidic properties, ensuring optimal detection of TAN-related compounds. The instrument was operated under optimized conditions to ensure high-quality data acquisition. For detailed instrument parameters and acquisition settings, including detection range, ion accumulation, and source parameters, please refer to Supporting Information Text 1.

The data files were internally recalibrated using the DataAnalysis software (Bruker Daltonics, Bremen, Germany) based on selected internal reference peaks from the Kendrick homologous series. The mass spectra were then processed using Composer software (Sierra Analytics, Modesto, USA) to assign molecular formulas to the detected ions.

Additional details regarding formula assignment in Composer, including constraints, mass error tolerance, and grouping based on heteroatom types, DBE, and carbon number, as well as the organization of data into composition tables for machine learning applications, are provided in Supporting Information Text 2.

### Multivariate Analysis

#### Data Pretreatment

The ESI (−) FT-ICR MS data utilized to build the models were extracted from the composition table generated from the Composer software and imported into MATLAB 2024a (MathWorks, Natick, USA). A data matrix (**X**) was created from the monoisotopic abundance values, also known as the independent variables. The rows of **X** represent the oil samples, and the columns represent the variables. As the acidity of the oil samples depends on different functional groups, all molecular formulas found in ESI (−) FT-ICR MS were initially considered. Then, each row of **X** was normalized by the sum of the intensities of all ions detected in each sample. Some strategies were applied to preselect the more relevant variables for modeling, such as excluding **X** columns with more than 25 zeros removed, as they were not informative.

#### Modeling

Multivariate data analysis was performed using Matlab 2024a software (MathWorks, Natick, USA). A vector containing the respective TAN values was built and designated **y**, the dependent variable. The **y** vector has rows equal to the number of samples in matrix **X**. The **y** vector differed depending on the TAN values modeled, i.e., TAN values for crude oil, jet fuel, diesel, gas oil, and vacuum residue. However, the **X** matrix was the same for all **y** vectors, i.e., the matrix **X** related to the crude oil was used to predict the TAN values of the crude oil itself and for its distillation fractions.

Partial least squares (PLS) is a regression method widely used in multivariate analysis.^35,36^ This method is proper when variables are highly correlated and when the number of independent variables (columns of matrix **X**) exceeds the number of samples (rows of matrix **X**). FT-ICR MS data are inherently high-dimensional and exhibit strong multicollinearity, which PLS effectively addresses by reducing dimensionality while maintaining focus on the response variable. This method was used for their ability to balance predictive performance and interpretability, which is critical in this study for understanding the relationship between molecular features and TAN.

The **y** vector was always mean-centered during preprocessing to focus the model on variations relative to the mean value, improving numerical stability and interpretation. Various preprocessing methods, such as L1 norm, L2 norm, infinity norm, and autoscale, were applied to the rows of matrix **X** to find the best prediction model. Details on these preprocessing methods are provided in Supporting Information Text 3.

The set of 36 samples was randomly divided into a calibration set (25 samples used to build the model) and an external validation set (11 samples used to validate the model built). During model development, 10-fold cross-validation was applied within the calibration set to optimize the number of latent variables (NLV) and evaluate the model performance. This procedure minimized the risk of overfitting by ensuring that the calibration process was robust and generalizable. External validation was subsequently performed to test the model’s predictive ability on unseen data. The performance of the models was assessed by the root-mean-square error (RMSE) and the correlation coefficient (*R*). Details of the optimization process are provided in Supporting Information, Text 4.
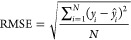
1
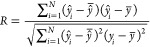
2where *y*_*i*_ and *ŷ*_*i*_ are the measured and predicted values, respectively, of a given sample *i*. The variables *y̅* and  are the means of the measured and predicted values for a set of *n* calibration samples. For calibration samples, *N* represents the number of samples in the calibration set, and the correlation and error are called the correlation coefficient of calibration (Rc) and the root-mean-square error of calibration (RMSEC), respectively. For external validation, *N* represents the number of samples in the prediction set, and the correlation and error are called the correlation coefficient of prediction (Rp) and the root-mean-square error of prediction (RMSEP), respectively.

#### Outlier Detection

Outlier detection was conducted using several diagnostic plots and statistical tests^37−39^ to ensure the robustness and accuracy of the PLS models. Leverage and studentized residual plots were employed to investigate potential outliers. Additionally, Hotelling’s T^2^ statistic and Q residuals were used as complementary tools for outlier detection.^40,41^

#### Variable Selection

Feature selection was conducted using the ordered predictors’ selection (OPS) algorithm.^28,29^ OPS involves sorting variables based on informative vectors and systematically exploring regression models to identify the most relevant set of variables. The approach *feedOPS* was used considering all of the available informative vectors. The variables were searched using a window of 10 and an increment of 2. Additional information can be found elsewhere for a more in-depth understanding of the OPS algorithm.^29^ Hereafter, PLS is used to represent the models that used all variables available, and PLS-OPS is used to represent the models that used the variables selected using the OPS.

## Results and Discussion

### Samples Characterization

The application of negative ESI, as demonstrated in previous studies, enabled the selective detection of acidic species in the 36 crude oil samples, aligning seamlessly with the study’s goal of identifying molecular contributors to the TAN. The high quality of the FT-ICR MS data is evidenced in Table S1 (Supporting Information), which details the number and percentage of peaks successfully assigned to molecular formulas, while Figure S1 (Supporting Information) presents the error distribution across the *m*/*z* range, further validating the reliability and precision of the mass spectrometric analysis.^8,22,42^ The 36 crude oil samples were initially characterized by analyzing their spectra, as presented in Figure S2 of the Supporting Information. This comprehensive spectral analysis provides an overview of each sample’s chemical composition, highlighting the diversity within the data set. Following the spectral analysis, the class distribution of the samples is presented, initially categorized into 20 classes, as shown in Figure S3 of the Supporting Information. This extensive classification underscores the variability and complexity of the crude oil samples.

**Figure 1:**
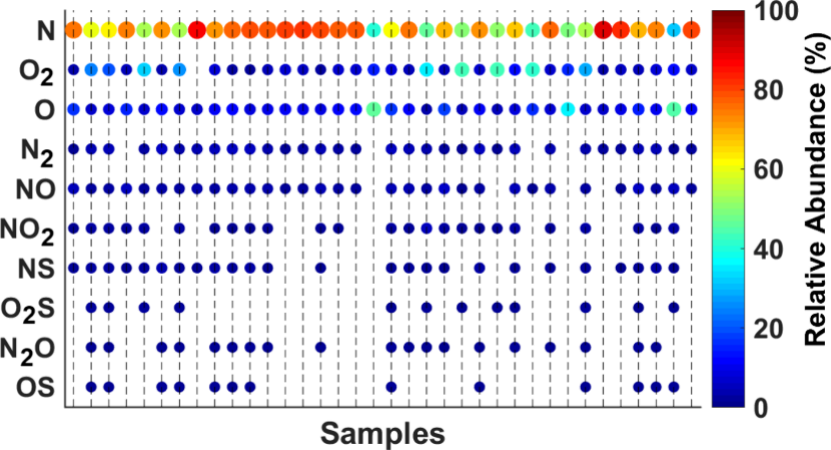
Class distribution of 10 compound classes selected during the preselect variables step before modeling. The plot shows the relative abundance (%) of various nitrogen (N, N_2_), nitrogen–oxygen (N_2_O, NO, NO_2_), nitrogen–sulfur (NS), oxygen (O, O_2_), oxygen–sulfur (O_2_S), and oxygen–sulfur (OS) containing compounds across the samples.

Furthermore, the class distribution was refined to 10 classes, selected during the preselection of variables before modeling. Classes with relative abundances of >1% in at least 50% of the samples and >5% in all samples were selected for modeling. This refined selection is presented in Figure 1, demonstrating a simplified yet effective classification that retains the essential variability of the samples.

Figure 1 presents a detailed visualization of the relative abundance of various compound classes across 36 crude oil samples. The *x*-axis displays the individual samples, with each vertical line representing a single sample. The *y*-axis lists the different compound classes, ordered from the most abundant on average to the least abundant. The color scale and size of the circles both correspond to the relative abundance (%) of each compound class within a sample. Warmer colors (ranging from yellow to red) and larger circles indicate higher relative abundances, while cooler colors (ranging from green to blue) and smaller circles represent lower relative abundances. Empty spaces in the grid indicate the absence of a particular compound class in that sample.

Compounds containing only one nitrogen (N) atom in their molecular formula have the highest relative abundance, with many data points close to 100%, indicating their dominance in the samples. In contrast, the classes of compounds such as N_2_, N_2_O, NO, NO_2_, NS, O_2_S, and OS show significantly lower relative abundances, with most data points clustered at the lower end of the scale (0–10%), indicating their presence in much smaller quantities. Compounds containing only oxygen (O and O_2_) also show moderate relative abundance, with a range of values that characterize their variable presence in the samples.

A notable observation is that two of the samples contain only three classes: N, which is the most abundant, followed by O and O_2_. These samples are easily identified by the presence of circles in only three rows corresponding to these classes, with the N class showing the largest and warmest-colored circles, indicating its dominance. Overall, this distribution provides a clear and comprehensive overview of compound classes across the crude oil samples, highlighting the variability in chemical composition and the dominance of certain classes in specific samples.

**Figure 2:**
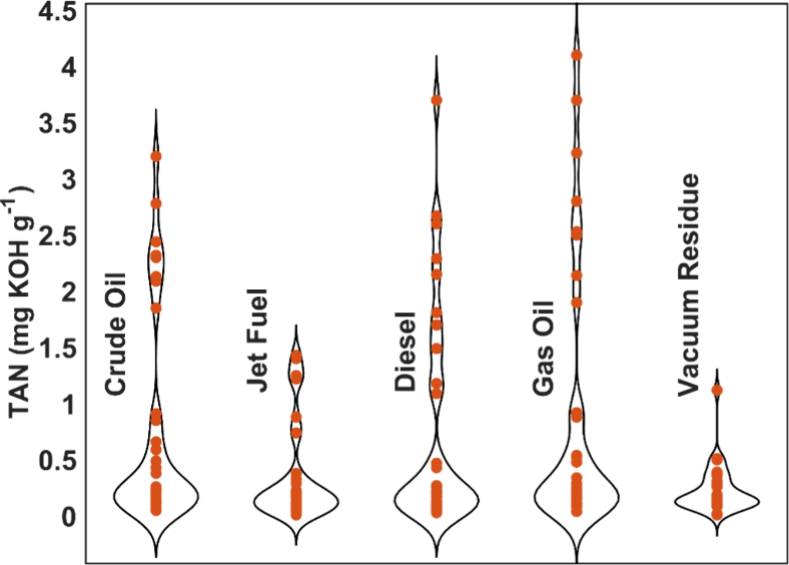
Violin plots of TAN values for petroleum-derived samples (crude oil and its distillation cuts: jet fuel, diesel, gas oil, and vacuum residue).

Figure 2 presents violin plots of the TAN values for crude oil and its distillation cuts, including jet fuel, diesel, gas oil, and vacuum residue. This visualization provides a comprehensive comparison of TAN distributions across different petroleum fractions.

Violin plots were used to illustrate the density and spread of the data. These representations combine a box plot and a kernel density plot to show the distribution and frequency of the TAN values. The width of each violin plot at any given TAN value corresponds to the frequency of that value within the data set. More comprehensive sections of the plot indicate higher frequencies of TAN values, while narrower sections indicate lower frequencies.

Crude oil exhibits a broad distribution of TAN values with significant variability, as indicated by the spread variation within the plot. Jet fuel shows a narrower distribution of TAN values, concentrated around lower values, reflecting its relatively lower acidity compared to other fractions. Diesel has a broader range of TAN values, suggesting more significant variability in its acidic properties. Gas oil displays the highest TAN values, with a considerable spread, indicating diverse acidic content within this fraction. Vacuum residue shows the lowest TAN values among the fractions, with a narrow distribution suggesting consistent low acidity.

**Figure 3:**
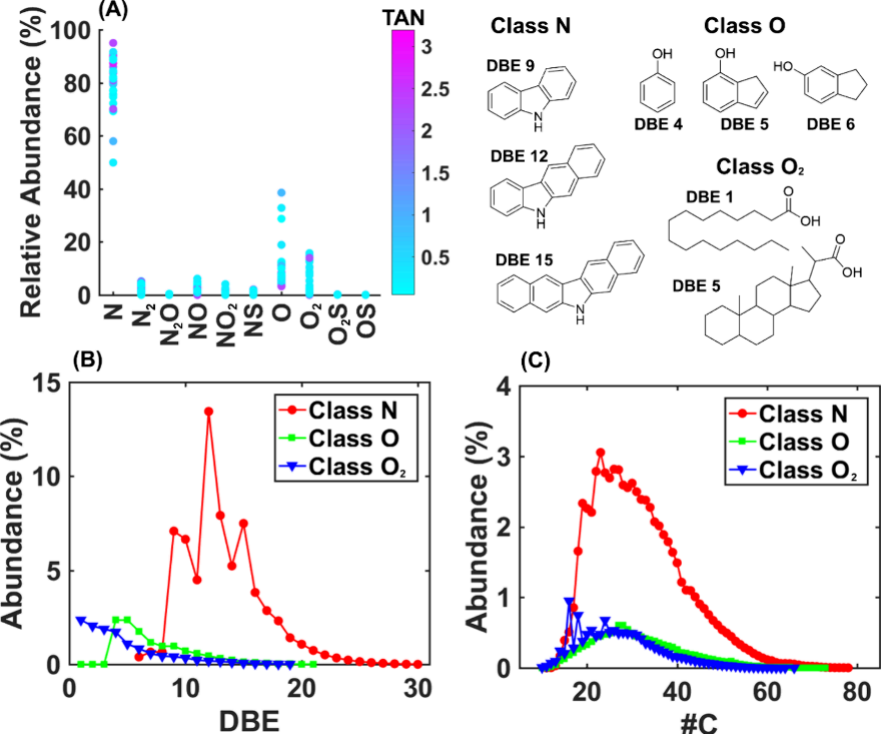
(A) Distribution of compound classes across 36 crude oil samples, combined with the TAN values (mg of KOH g^–1^ oil) represented by the color scale. The most prominent classes (N, O, and O_2_) are highlighted for their significant variation in relative abundance and TAN values. (B) Distribution of double bond equivalents (DBE) for the three most abundant classes (N, O, and O_2_), showing the representative DBE values for these compounds across the samples. (C) Distribution of the number of carbon atoms (#C) for the same classes. The molecular structures correspond to the most abundant core structures within the three classes.

Figure 3A presents the distribution of compound classes across 36 crude oil samples combined with the crude oil TAN values displayed on the color scale. The figure highlights the relative abundance of different compound classes and their relationship with TAN values. The most prominent classes, particularly N, O, and O_2_, show significant variation in relative abundance and TAN, making them key contributors to the overall acidity of the samples. Figure 3B,C further explore the distribution of double bond equivalents (DBE) and the number of carbon atoms (#C), which are representative of the samples from these three most abundant classes.

The visualization in Figure 3A clearly indicates that classes N, O, and O_2_ are the most influential in determining the TAN values across the samples. The strong correlation between higher TAN values and the relative abundance of these classes suggests that they are critical in driving the acidity of crude oil.^7,20^ The detailed molecular structures shown in the inset further underscore the variety within these classes, particularly concerning DBE, which correlates with molecular unsaturation and aromaticity.

Figure 3B,C provide further insight by showing the distribution of DBE and #C for the N, O, and O_2_ classes, which are representative of these samples. The peaks in DBE for the N class indicate the importance of aromatic and polycyclic nitrogen compounds, specifically carbazoles and their benzohomologues, in contributing to TAN.^8^ Carbazoles are tricyclic compounds that contain nitrogen and are known for their stability and aromaticity. The DBE values of 9, 12, and 15 correspond to carbazoles and their extended structures with additional aromatic rings. These compounds are significant contributors to TAN because their nitrogen atoms can increase the acidity of crude oil, especially when these structures are present in higher concentrations.

For the O class, a notable observation is the presence of phenolic compounds, which have DBE values starting at 4. These compounds are typically characterized by a single aromatic ring with one hydroxyl group attached. The relatively low DBE of 4 corresponds to the basic structure of phenols, which are known for their acidic properties and can significantly contribute to the overall TAN of crude oil.^43^

The O_2_ class, which includes compounds with two oxygen atoms, is primarily characterized by the presence of linear saturated acids.^6,8,20^ The #C distributions (Figure 3C) reveal peaks at 16 and 18 carbon atoms, corresponding to long-chain fatty acids, such as palmitic acid (C16) and stearic acid (C18). However, the O_2_ class also includes saturated and aromatic acids with ring structures. These acids, while less prevalent than the linear saturated acids, also contribute to the overall TAN. The combination of these different types of acids within the O_2_ class underscores the complexity of this group and its significant impact on the acidity of the crude oil samples.

To further investigate the relationship between variable classes and TAN values in crude oil, the samples with low (∼0.06 mg KOH g^–1^ oil) and high (∼2.3 mg KOH g^–1^ oil) TAN values were compared using volcano plots (Figure S4, Supporting Information). The comparisons were made separately for the three most prominent classes: N, O, and O_2_. The volcano plot results showed a higher fold change for compounds with O_2_ in samples with higher acidity and for compounds with N and O in samples with lower acidity. Further explanations are provided in Supporting Information Text 6.

In summary, the characterization of crude oil samples through the analysis of compound classes and their relationship with TAN values has provided valuable insights into the chemical contributors to acidity in these samples. The detailed examination of class N and oxygen-containing compounds (classes O and O_2_) highlights the significant roles that these classes play in determining TAN. Notably, the distribution patterns observed in the volcano plots and DBE analysis underscore the impact of specific structures, such as naphthenic acids, in driving the acidity levels. This comprehensive characterization sets a strong foundation for the subsequent development of regression models, which will leverage these insights to predict TAN values more accurately and efficiently across different crude oil samples.

### Regression Models

**Figure 4:**
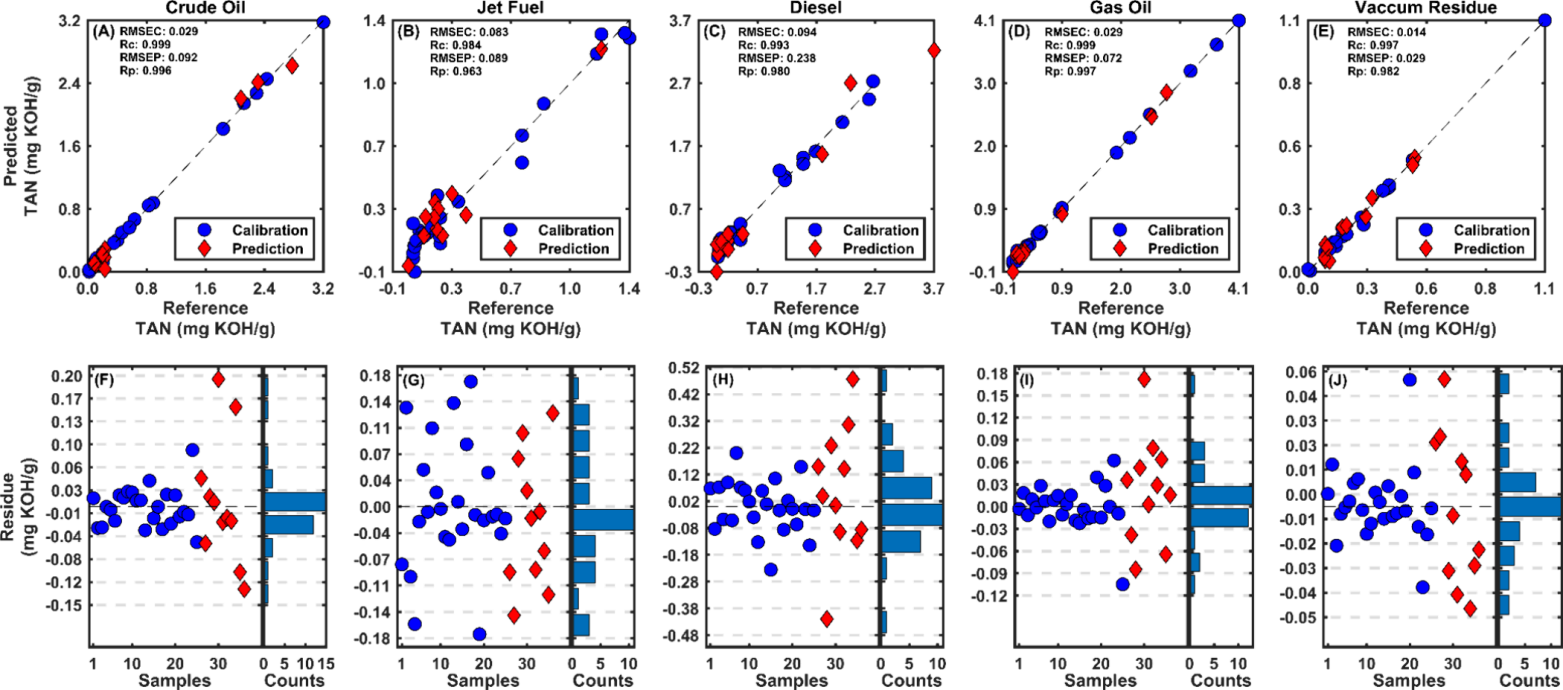
Comparison of measured reference and predicted TAN values for crude oil and its distillation cuts [(A) crude Oil, (B) jet fuel, (C) diesel, (D) gas oil, and (E) vacuum residue]. The top row shows scatter plots of predicted versus reference TAN values, with the dashed line representing the ideal 1:1 correlation. The bottom row presents the residuals for the predictive models of TAN values in (F) crude oil, (G) jet fuel, (H) diesel, (I) gas oil, and (J) vacuum residue. The blue circles represent calibration samples, and the red diamonds represent prediction samples. The histograms on the right side of each plot show the distribution of residuals, providing a visual assessment of the residuals’ spread and central tendency.

In this section, we present the results of the regression models developed to predict the TAN of crude oils and their distillation cuts based on the compositional data obtained from the FT-ICR MS spectra of crude oil. The detailed results are presented in Table S2 with the performance of PLS and PLS-OPS models. Figure 4 showcases the performance of the PLS-OPS regression models by comparing the predicted TAN values with the measured reference values for crude oil and its fractions. Each subplot in the top row represents a different fraction with the *x*-axis showing the reference TAN values and the *y*-axis showing the predicted TAN values. The points closely aligned with the diagonal dashed line indicate a strong agreement between the predicted and actual values, demonstrating the accuracy of the models. Additional details regarding model optimization are provided in Figures S5 and S6 (Supporting Information). Figure S5 illustrates the RMSECV as a function of the NLV, highlighting the process used to optimize the model structure. Figure S6 shows the evolution of variable selection through OPS, demonstrating how the number of selected variables impacts the cross-validation error during the internal validation and model-building process.

For crude oil, both PLS and PLS-OPS exhibit reasonably low RMSEC values, indicating effective model fitting, with PLS-OPS demonstrating notable improvement in the Rc and Rp. This suggests that the OPS enhances the model’s accuracy in capturing the variation in TAN for crude oil. Similar trends are observed for jet fuel, diesel, gas oil, and vacuum residue, where PLS-OPS consistently outperforms the standard PLS method regarding Rc and Rp, showcasing its efficacy in variable selection for improved model performance.

Furthermore, the number of variables (Table S2, Supporting Information) selected by PLS-OPS is considerably reduced compared to using all variables in PLS, demonstrating the efficiency of the OPS method in identifying key variables relevant to TAN prediction. The results also highlight the application of PLS-OPS in overcoming overfitting issues, as evidenced by the lower RMSEP values in PLS-OPS models. These findings underscore the importance of employing variable selection techniques such as PLS-OPS to enhance the robustness and interpretability of predictive models for TAN in diverse oil fractions.

The relative errors (Figure S7, Supporting Information) reveal some instances of high relative errors that appear disproportionately large. These high relative errors might seem unacceptable for a predictive model at first glance, especially when the typical expectations for model accuracy. This discrepancy raises concerns and warrants closer examination.

In predictive modeling, such high relative errors can occur even when the predicted values are numerically close to the measured values, particularly when the actual TAN values are very low. In such cases, even small absolute differences between the predicted and measured values can result in large relative error percentages. This phenomenon is particularly evident in samples with very low TAN values, where the relative error can be misleadingly high despite the model’s strong overall performance.

Given these observations, a thorough investigation of all samples was conducted to identify potential outliers. Each sample with a high relative error was scrutinized to determine whether it represented an anomaly or if the model’s prediction was systematically off for certain types of samples.

Despite the initial concern raised by the high relative errors in some samples, particularly in those with low TAN values, the investigation revealed that none of these samples could be definitively classified as outliers. The model provides reliable predictions for higher TAN values, addressing critical industrial needs while acknowledging that accuracy for low TAN samples could be improved with future refinements.

The leverage and studentized residual plots (Figure S8, Supporting Information) did not indicate any sample with a leverage value far exceeding the others, nor did the studentized residuals suggest any extreme deviations. Similarly, Hotelling’s T^2^ and Q residuals (Figure S8, Supporting Information) were within acceptable ranges, further supporting that these samples were consistent with the overall data set and did not compromise the model’s integrity. Consequently, no samples were removed from the analysis, as they were determined to be valid data points rather than outliers. The decision to retain all samples was further supported by the results of the Shapiro-Wilk test [*p* values presented in Table S3 (Supporting Information)], which was applied to the residuals of the predictions.

Following this analysis, residuals provide a more faithful representation of model robustness than relative error alone. By examination of the distribution of residuals (Figure 4), one can assess how well the model captures the underlying trends in the data. In this case, the residuals followed a normal distribution, as validated by the Shapiro–Wilk test with a 95% confidence level. The histogram of the residuals, displayed alongside the normal distribution, further supports this conclusion. The close alignment of the residuals with a normal distribution suggests that the predictive models are both reliable and accurate, reinforcing their utility for determining TAN values across crude oil and its distillation cuts. Table S3 (Supporting Information) presents the means and standard deviations of the residuals, along with the corresponding values for 2 and 3 times the standard deviation (2σ and 3σ), which represent 95 and 99% confidence intervals, respectively. For instance, in the case of the crude oil model, the standard deviation of the residuals is 0.018 mg KOH/g. This indicates that approximately 68% of the residuals are expected to fall within the range of ±0.018 mg KOH/g from the mean (1σ). When considering 2 times the standard deviation, corresponding to ±0.037 mg KOH g^–1^ oil, about 95% of the residuals should lie within this interval (2σ). Finally, with 3 times the standard deviation, we obtain a range of ±0.055 mg KOH g^–1^ oil, covering 99% of the residuals (3σ).

Therefore, for the crude oil model, we can assert that 99% of the TAN predictions have an expected residual error within ±0.055 mg of KOH g^–1^ of oil, based on the performance observed in the calibration set. This parameter serves as a confidence measure in the model’s accuracy, allowing for an assessment of how well the TAN predictions align with the measured values. Table S3 includes similar data for the distillation cut models, providing a comprehensive view of the models’ robustness under different conditions.

While the regression models demonstrated strong performance based on the calculated metrics, it is important to acknowledge the limitations associated with the relatively small data set of 36 samples. Although rigorous cross-validation techniques were employed to optimize the models and mitigate the risk of overfitting, the small sample size could affect the generalizability of the results to broader data sets. Future work should focus on expanding the data set to include additional crude oil samples and distillation fractions with varying chemical compositions. This expansion would not only enhance model robustness but also allow for a more comprehensive exploration of the relationship between molecular features and TAN, potentially uncovering new insights into crude oil chemistry.

After establishing the models’ performance, it is crucial to understand the types of variables selected by the OPS responsible for the final prediction accuracy. By analyzing the variables selected by the OPS, we can gain insights into which chemical components or classes in the crude oil spectra are most influential in determining TAN values.

### Variable Selection

**Figure 5:**
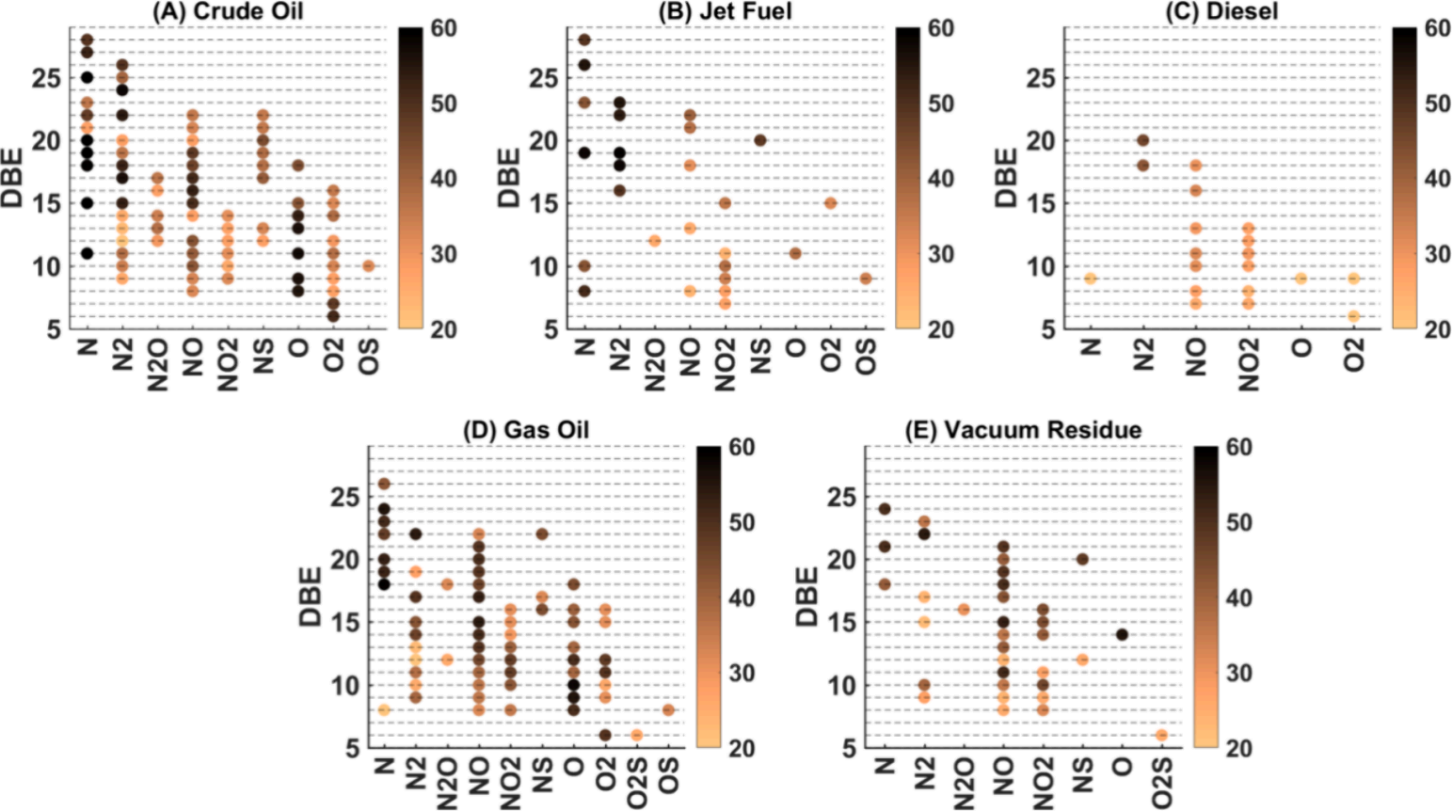
Selected variables by the OPS method highlighting double bond equivalents (DBE) and the number of carbon atoms (#C) for each modeled class: crude oil, jet fuel, diesel, gas oil, and vacuum residue. (A) Crude oil, (B) jet fuel, (C) diesel, (D) gas oil, and (E) vacuum residue. The color scale indicates the #C of the selected variables.

Figure 5 presents the selected variables concerning DBE on the *y*-axis, with the color scale representing the number of carbon atoms for each modeled class of crude oil and its distillation cuts.

The selected variables across the models for crude oil and its distillation cuts (jet fuel, diesel, gas oil, and vacuum residue) reveal significant diversity in both the DBE and the number of carbon atoms. The DBE values span a broad range, generally between 5 and 25, indicating the presence of both saturated and unsaturated structures across all fractions. This range reflects the necessity of accounting for various degrees of unsaturation to accurately predict the TAN in each fraction.

Molecules from classes N, O, and O_2_ are consistently prominent among the selected variables across all models. Notably, the O_2_ class was not selected in the vacuum residue model, highlighting some variability in the importance of specific oxygenated species across different fractions. Additionally, nitrogen and nitrogen–oxygen compounds, such as N_2_, NO, and NO_2_, also appear as significant contributors in several models, underscoring their relevance in predicting TAN.

Among the nitrogen-containing species, the N_2_ class is particularly noteworthy. The N_2_ class species with a DBE of 17 likely corresponds to molecules with two fused carbazole cores. Additionally, the series with a DBE of 18 could represent structures such as a benzocarbazole fused with a quinoline molecule or two carbazoles joined by a bridge bond.^44^ The NO_*x*_ classes (NO and NO_2_) are also significant in the selected variables. The NO class, which was observed across multiple fractions, likely corresponds to products derived from the biodegradation process, where carboxylic acid is added to pyrrole nuclei, forming complex nitrogen–oxygen structures.^8^ These compounds may play a role in differentiating TAN values by introducing variations in acidity due to their unique chemical properties. Additionally, the broader NO_*x*_ class may translate to furrolic, phenolic, and/or carboxylic analogues,^19^ which are also important in determining the TAN.

The number of carbon atoms in these selected variables typically ranges from 20 to 60, demonstrating that molecules of moderate to large sizes play a significant role in the predictive models for all oil fractions. The complexity and diversity of each fraction are further highlighted by the distributions of these variables. For example, the vacuum residue, which is the heaviest fraction, requires a more extensive and varied set of predictors, as evidenced by the broader range of DBE and #C values. This reflects the intricate nature of this fraction, where both moderately and highly unsaturated species as well as a wider range of molecular sizes are crucial for accurate TAN prediction.

Overall, the selected variables across all fractions highlight the varying degrees of complexity and the necessity of a tailored set of predictors for each fraction. With their more complex profiles, crude oil and gas oil require a diverse set of variables spanning wide DBE and #C ranges. In contrast, with a simpler composition, jet fuel needs fewer and fewer complex predictors. Diesel and vacuum residue fall in between, reflecting their moderate to high complexity. Understanding these key contributors helps refine predictive models and supports more efficient processing and quality control across different fractions, ensuring accurate TAN determination and optimizing refining processes.

### TAN Boiling Point Distribution

**Figure 6:**
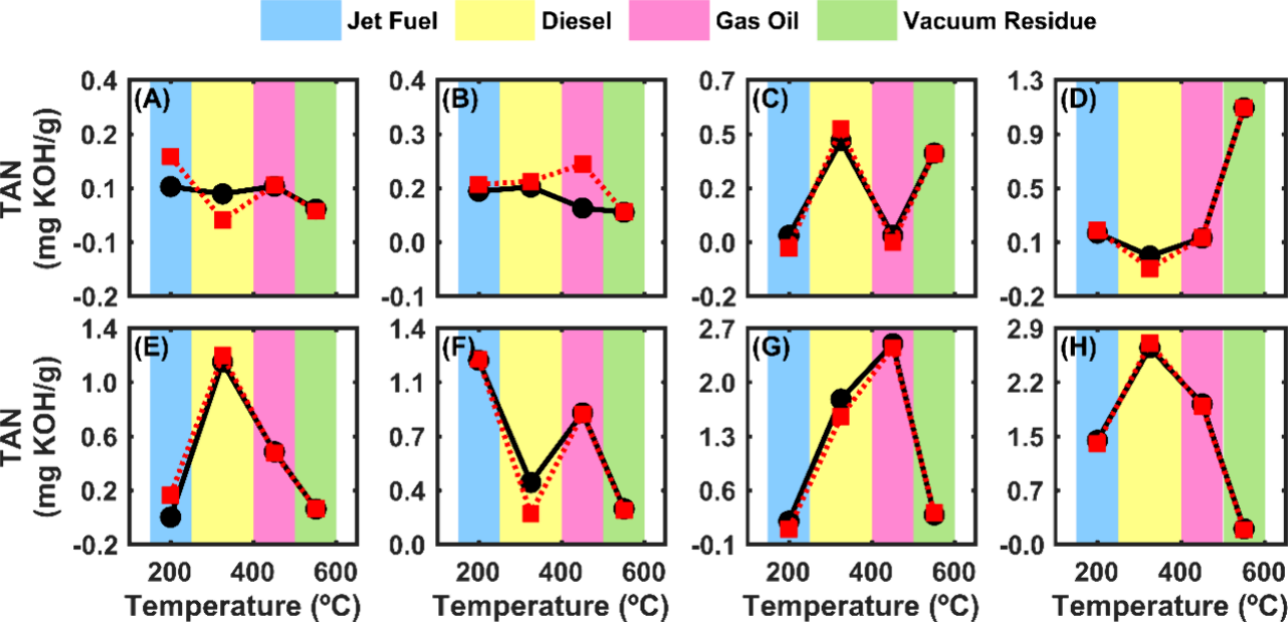
TAN true boiling point (TBP) distribution for eight crude oil samples (A–H) with varying TAN values across different distillation cuts. The *x*-axis represents the approximate average temperature range corresponding to the boiling points of the cuts: jet fuel (150–250 °C), diesel (250–400 °C), gas oil (400–500 °C), and vacuum residue (500–600 °C). The black solid line represents the reference TAN values measured by titration, while the red dashed line shows the TAN values predicted by the model.

TAN and its distribution as a function of the true boiling point (TBP) are crucial parameters for understanding the acidic content of crude oils and their distillation cuts. This distribution, often termed the TAN true boiling point distribution, provides insight into the TAN values across different boiling ranges. The TAN TBP distribution is essential for optimizing refinery operations, managing corrosion, and ensuring the safe processing of high-TAN crudes. Traditionally, as mentioned earlier, the TAN is determined through nonaqueous titration, and the BP distribution is obtained by measuring the TAN in selected distillation cuts. This process often involves interpolation and extrapolation to estimate the TAN across the boiling range.^45^ However, the traditional method is time-consuming, requiring physical distillation of the crude, followed by titration of the cuts, making the development of more efficient analytical methods a priority for the industry. In this work, since we developed predictive models for TAN in crude oil and its distillation cuts using only crude oil samples, we are able to obtain the TBP distribution of the cuts directly, as shown in Figure 6.

The TAN TBP distribution curves presented in Figure 6 demonstrate a close alignment between the reference TAN values (black solid lines) and the predicted TAN values (red dashed lines) across the different distillation cuts. This alignment suggests that the predictive model performs well in capturing the distribution of acidity within the crude oil samples. Notably, as the TAN values increase from the sample shown in Figure S9A–H, the agreement between the predicted and reference curves improves, indicating that the model’s accuracy is particularly robust for samples with higher TAN values. A broader overview of the TAN TBP distribution for all 36 samples is provided in Supporting Information Figure S9, further confirming the model’s consistency across a diverse range of crude oils.

In summary, the method developed in this study offers an innovative and efficient approach for predicting the TAN of crude oils and their distillation cuts, utilizing exclusively FT-ICR MS. This approach represents a significant advancement in petroleomics, as it enables the detailed assessment of petroleum acidity without the need for the time-consuming and costly processes traditionally required, such as physical distillation and titration. The key advantage of this method is its ability to transform a single mass spectrometry analysis into an accurate prediction of complex properties, such as the TAN in crude oil distillation cuts. This capability is particularly relevant for the petroleum industry, where critical concerns include optimizing refinery operations, managing corrosion risks, and safely processing high-TAN crudes. Beyond TAN prediction, the integration of FT-ICR MS data with predictive modeling approaches has the potential to be expanded to other key applications. For example, nitrogen- and sulfur-containing compounds, which play critical roles in determining crude oil quality and environmental compliance, have already been explored by our research group. These studies highlight the versatility of this approach in predicting properties linked to the molecular composition.

Furthermore, the methodology could be extended to address asphaltene deposition, which is a significant issue in refining and transportation processes. Predictive models capable of correlating FT-ICR MS data with asphaltene deposition tendencies could provide valuable insights for preventing pipeline blockages and optimizing refining strategies. This flexibility underscores the broader applicability of this approach to tackling a range of challenges associated with crude oil and its fractions. The application of FT-ICR MS, with its ultrahigh resolution and accuracy, allows for comprehensive characterization of the complex mixtures found in crude oil, enabling the identification and quantification of thousands of molecular species simultaneously.

Technological advancements in mass spectrometry, particularly through FT-ICR MS, have been instrumental in realizing this method, making it a valuable tool for the industry. Accurate prediction of TAN using this method not only conserves time and resources but also provides critical insights that can be utilized to optimize refinery operations and ensure safety in the processing of crude oils with varying levels of acidity.

This work clearly demonstrates the potential of FT-ICR MS to transform the determination of critical petroleum properties, opening new avenues for the application of petroleomics in industry and other areas that handle complex mixtures. As mass spectrometry technology continues to evolve, the potential for its application in petroleomics will only expand, driving further innovation and optimization in crude oil processing.

## Conclusions

This study successfully applies PLS and OPS models to predict TAN in crude oil and its distillation cuts, specifically in true boiling point distributions, using ultrahigh resolution mass spectra directly from crude oil composition, without the need for further distillation. The development and validation of these models demonstrate robust predictive performance, evidenced by low RMSEs and high correlation coefficients, while eliminating the need for additional time-consuming assays, such as the distillation ASTM protocols, further underscoring their accuracy and efficiency in forecasting TAN. The initial characterization of the crude oil samples provided a comprehensive overview of their chemical composition. Spectral analysis and class distribution revealed the diversity and complexity inherent to the samples. By grouping the samples based on TAN values and conducting paired analyses using volcano plots, we identified significant variable classes associated with changes in TAN values. This detailed analysis highlighted the dynamic relationship between TAN values and the abundance of specific compound classes, emphasizing the critical role of nitrogen- and oxygen-containing compounds.

The models’ results showed a strong agreement between predicted and measured TAN values and the minimal residuals, which further confirmed the models’ robustness. A thorough evaluation of potential outliers was conducted, and it was determined that no samples needed to be removed, as even those with higher relative errors did not unduly influence the model’s performance. The analysis of selected variables by OPS provided more profound insights into the specific chemical characteristics driving the TAN predictions. The selected variables varied significantly across different fractions, reflecting the diverse and complex nature of the samples. Furthermore, the TAN TBP distribution analysis showed a strong correlation between predicted and reference curves, especially as TAN values increased, demonstrating the method’s applicability for refining processes.

The quality of the FT-ICR MS data, including precise calibration and accurate molecular formula assignment, was critical in ensuring the reliability of the models’ predictions. These foundational aspects highlight the robustness and reproducibility of the analytical workflow. Additionally, the approach demonstrated in this study has the potential for broader applications beyond TAN prediction. For instance, the same methodology could be adapted to predict properties such as asphaltene deposition tendencies or sulfur compound distributions, both of which are critical for refining operations and environmental compliance.

In conclusion, this research highlights the efficacy of combining advanced mass spectrometry techniques with machine learning models to predict TAN values accurately. The strong predictive performance of the models and the detailed understanding of the key chemical contributors provide valuable tools for the petroleum industry. These insights can significantly streamline the process of TAN determination, facilitating more efficient refining processes and better quality control. This study paves the way for further advancements in predictive modeling and analytical techniques in the context of complex sample properties.

## Supporting Information Available

Comprehensive details that complement the main manuscript, additional texts covering mass spectrometry analysis, data processing and organization, data pretreatment methods, model optimization, outlier detection, and volcano plot analysis, summary of the percentage and number of assigned peaks for all samples, model parameters, and the results of statistical tests on residuals, error distribution of *m*/*z* values for all samples, mass spectra, and the complete class distribution of all samples, as well as volcano plots showing significant differences in TAN-related molecular classes, additional modeling optimization plots, including RMSECV versus the number of latent variables and OPS variable selection curves, relative error plots, leverage versus residual plots, Hotelling’s T^2^ versus Q residual plots, and TBP distribution plots for all samples, and comprehensive set of supporting data that ensures the transparency and reproducibility of the methods and results presented in this study (PDF)
