# Supplementary material for: Integrating FT-ICR MS and Machine Learning to Forecast Acid Content Across Boiling Cuts
Source: Anal Chem. 2025 Feb 26;97(11):5965–74. doi: 10.1021/acs.analchem.4c04522 (PMC11948185; doi:10.1021/acs.analchem.4c04522)
Supplement: Supplementary file 1 — ac4c04522_si_001.pdf [file ac4c04522_si_001.pdf]

Supporting Information

Integrating FT-ICR MS and Machine Learning to Forecast Acid Content Across Boiling Cuts

Jussara V. Roque[1#], Wilson J. Cardoso[1], Deborah V. A. de Aguiar[1], Gabriel F. dos Santos[1], Alexandre de O. Gomes[2], Iris Medeiros Júnior[2], Gesiane da S. Lima[1#*], Boniek Gontijo [1*]

[1] Laboratory of Chromatography and Mass Spectrometry, Institute of Chemistry, Federal University of Goiás, 74690-900, Goiânia, GO, Brazil

[2] CENPES, PETROBRAS, 21941-915, Rio de Janeiro, RJ, Brazil.

Corresponding authors

E-mail address: *lima.gesiane12@gmail.com; *boniek@ufg.br.

[#]Equally contributed to this work.

Table of Contents

Text 1 – Mass Spectrometry Analysis S2
Text 2 – Data Processing and Organization S3
Text 3 – Data Pre-treatment S3
Text 4 – Optimization and Modeling S4
Text 5 – Outliers Detection (Experimental) S5
Text 6 – Volcano plots S6
Table S1.. .... S8
Table S2. .... S9
Table S3. .... S10
Figure S1. .... S12
Figure S2. .... S13
Figure S3. .... S14

Figure S4. ....S16
Figure S5. ....S16
Figure S6. ....S17
Figure S7. ....S18
Figure S8. ....S20
Figure S9. ....S21

Text 1 – Mass Spectrometry Analysis

Data sets of 8 megawords were acquired through the magnitude mode with a detection range of m/z 150-2000 in negative-ion mode. A total of 300 scans with an ion accumulation of 0.02 s were accumulated to obtain spectra with excellent signal-to-noise values. Samples were directly infused at a flow rate of 120 μL h-1. Source parameters were set as follows: dry gas (nitrogen) flow rate 1 L min^{-1} at 200 °C, capillary voltage 3.8 kV, and spray shield voltage −500 V.

Text 2 – Data Processing and Organization

Data Processing: The data processing involved internal recalibration using Kendrick homologous series in DataAnalysis software (Bruker Daltonics). Molecular formula assignment was carried out in Composer software (Sierra Analytics, Modesto, USA) using hydrocarbon rules and the walking recalibration equation. The analysis was conducted within an *m/z* range of 150–2000, with a double bond equivalent (DBE) range of 0–40, and a mass error tolerance of 0.7 ppm. The constraints applied allowed for up to 200 carbon atoms, 400 hydrogen atoms, 4 nitrogen atoms, 4 oxygen atoms, and 4 sulfur atoms per molecular formula.

Data Organization and Machine Learning Preparation: Processed data were grouped based on heteroatom types, hydrogen deficiency (DBE), and alkylation degree (carbon number). Composition tables generated by Composer software served as the foundational datasets for machine learning model development.

Text 3 – Data Pre-treatment

Pre-Processing Methods for Vector **y**

Mean-centering is a pre-processing step where the mean value of the response variable is subtracted from each observation. This transforms the vector to have a mean of zero, which enhances numerical stability and facilitates the interpretation of the regression coefficients by centering the predictions around the mean response.

<u>Pre-Processing Methods for Matrix **X**</u>

L1 Norm: This method calculates the absolute value of the area "under the curve," representing the sum of the absolute values of all data points.

L2 Norm: The L2 norm computes the squared values and represents the multivariate vector "length" or Euclidean norm.

Infinity Norm: This approach determines the maximum absolute value in each row.

Autoscale: Autoscale involves mean-centering each variable (subtracting the mean) and scaling it by its standard deviation. This ensures that the variables have a mean of zero and a standard deviation of one, placing them on a comparable scale and improving the performance of distance-based models.

Text 4 – Optimization and Modeling

The dataset of 36 samples was randomly divided into two subsets: a calibration set (25 samples) for model development and an external validation set (11 samples) for independent evaluation of model performance. Within the calibration set, 10-fold cross-validation was conducted to optimize the number of latent variables (NLV). The dataset was randomly partitioned into ten subsets, with nine subsets used for calibration and one subset for validation in each iteration. This

process was repeated ten times, ensuring that every subset served as the validation set once. Metrics such as root mean square error of calibration (RMSEC) and validation (RMSECV) were calculated to identify the optimal model parameters. Once the model was finalized, the external validation set was used to assess its predictive ability on unseen data. Metrics such as the root mean square error of prediction (RMSEP) and the correlation coefficient (R) were used to evaluate the model's performance. To ensure overfitting was avoided, we monitored the consistency between the calibration and external validation results, with no significant discrepancies observed. This dual approach provided a reliable framework for validating the models, ensuring both robustness and generalizability.

Text 5 – Outliers Detection (Experimental)

Leverage and Studentized Residuals

The leverage plot assesses the influence of each observation on the model, with higher leverage values indicating observations that have a greater impact on the model's predictions. The studentized residuals, which are residuals scaled by their estimated standard errors, provide insight into how well the model predicts individual data points. Values deviating significantly from the expected range may indicate outliers that could distort the model.

Hotelling's T^2 and Q residuals

Hotelling's T^2 statistic measures the distance of each observation from the center of the multivariate data distribution, with higher values indicating potential outliers in the predictor space. Q residuals, on the other hand, assess the distance between the actual data points and the model's predictions, identifying

observations that do not fit well with the model. Moreover, the Shapiro-Wilk test was applied to the residuals of the prediction with a 95% confidence level to assess the normality of the residuals. This test is crucial for validating the assumptions underlying the regression models, ensuring that the residuals are normally distributed. This is a key requirement for the validity of many statistical inference procedures.

Text 6 – Volcano plots

To complement the discussion in the manuscript on the relationship between variable classes and TAN values in crude oil, we performed a focused analysis comparing the samples with the lowest (~0.06 mg KOH g^{-1} oil) and highest (~2.3 mg KOH g^{-1} oil) TAN values using volcano plots. This analysis allows for a detailed examination of how specific compound classes (N, O, and O_2) differ between these acidity extremes. Figure S4 presents volcano plots for the three compound classes: N, O, and O_2. In these plots, each point represents a molecular formula, with the x-axis indicating the fold change (FC) between the two TAN groups and the *y*-axis representing the significance of the difference (−log10 *p*-value). Variables above the significance thresholds (delineated by vertical and horizontal lines) are considered significantly different between the two TAN groups. The volcano plots reveal notable findings for oxygen-containing compounds (classes O and O_2), as illustrated in Figures S4B and S4C. Specifically, Class O_2, which includes naphthenic acids, demonstrates a distinct distribution of elevated FC values to the right, indicating a strong correlation with high TAN levels. This observation reinforces the established understanding that naphthenic acids substantially contribute to the total acidity of crude oil. Additionally, the DBE distribution plot in Figure 3B shows that linear naphthenic

acids (DBE 1) are particularly influential. These compounds possess relatively low pKa values (ranging from 4 to 5), classifying them as moderately strong acids[1]. This acidity profile explains their significant contribution to increased TAN values in crude oil. This detailed analysis highlights the chemical specificity and relevance of oxygenated species, particularly naphthenic acids, in driving TAN variability across crude oil samples.

Table S1. Number of peaks assigned and % assigned for all 36 crude oil samples (P01-P36) analyzed by ESI (−) FT-ICR MS.

Crude oil samples	Number of peaks assigned	% Assigned	Crude oil samples	Number of peaks assigned	% Assigned
**P01**	99.67	3133	**P19**	98.99	3489
**P02**	99.70	2833	**P20**	99.36	3756
**P03**	99.72	3131	**P21**	99.22	4687
**P04**	99.28	4307	**P22**	99.62	3266
**P05**	99.59	3322	**P23**	99.66	3791
**P06**	99.41	3872	**P24**	98.96	3974
**P07**	99.38	4220	**P25**	99.86	1589
**P08**	99.35	4343	**P26**	99.87	1387
**P09**	99.70	3020	**P27**	99.85	1785
**P10**	98.85	2555	**P28**	99.85	1488
**P11**	99.36	3931	**P29**	99.92	1014
**P12**	99.44	4344	**P30**	99.88	1550
**P13**	98.55	2034	**P31**	99.89	1475
**P14**	98.75	3293	**P32**	99.69	1799
**P15**	98.94	3989	**P33**	99.87	867
**P16**	99.00	4269	**P34**	99.95	957
**P17**	99.77	2090	**P35**	99.85	1487
**P18**	99.45	3543	**P36**	99.93	1357

Table S2. Performance parameters of the PLS (all variables) and PLS-OPS models (variable selected by OPS) for different petroleum fractions.

Fraction	Method	Nvars	RMSEC	Rc	RMSECV	Rcv	RMSEP	Rp	NVL	PT
**Crude Oil**	PLS	3142	0.112	0.993	0.530	0.853	0.197	0.977	9	
	PLS-OPS	204	0.029	0.999	0.137	0.989	0.092	0.996		
**Jet Fuel**	PLS	3142	0.188	0.888	0.351	0.561	0.514	0.055	3	L1
	PLS-OPS	36	0.083	0.984	0.142	0.941	0.089	0.963		
**Diesel**	PLS	3142	0.297	0.930	0.520	0.767	0.815	0.969	5	
	PLS-OPS	68	0.094	0.993	0.134	0.985	0.238	0.980		
**Gas Oil**	PLS	3142	0.113	0.995	0.869	0.784	0.554	0.955	10	
	PLS-OPS	108	0.029	0.999	0.281	0.975	0.072	0.997		
**Vacuum Residue**	PLS	3142	0.038	0.986	0.162	0.696	0.110	0.155	9	L1
	PLS-OPS	42	0.014	0.997	0.063	0.956	0.029	0.982		

PLS: model using all variables; PLS-OPS: models using variables selected by OPS; PT: pre-processing; L1: L1 norm; NVL: number of latent variables; Nvars: number of variables in the model; RMSEC: root mean squared error of calibration; Rc: correlation coefficient of calibration; RMSECV: root mean squared error of cross-validation; Rcv: correlation coefficient of cross-validation; RMSEP: root mean squared error of prediction; Rp: correlation coefficient of prediction.

Table S3. Statistical analysis of the residuals for TAN prediction models across different oil fractions, including the mean residuals, standard deviations, and the corresponding ranges for 1σ (~68%), 2σ (~95%), and 3σ (~99%) confidence intervals. The table also includes the *p*-values from the Shapiro-Wilk test for normality of the residuals.

	**Mean**	**Standard deviation σ (~68%)**	**2σ (~95%)**	**3σ (~99%)**	***p*-value (Shapiro-Wilk test)**
**Crude Oil**	0.022	0.018	0.037	0.055	0.493
**Jet Fuel**	0.061	0.057	0.114	0.170	8×10^{-4}
**Diesel**	0.074	0.059	0.119	0.179	0.282
**Gas Oil**	0.019	0.022	0.044	0.066	0.377
**Vacuum Residue**	0.009	0.011	0.022	0.033	0.258

For most samples, the test confirmed that the residuals followed a normal distribution with 95% confidence, indicating that the model's assumptions were met and that the predictions were statistically sound. However, for the jet fuel model, the Shapiro-Wilk test indicated a significant *p*-value, suggesting that the residuals did not follow a normal distribution. In the case of jet fuel, one specific sample with the highest TAN value exhibited a different behavior. However, this is likely due to the lack for other samples with similarly high TAN values in the dataset, rather than it being an outlier. This interpretation is supported by the fact that when the model was reconstructed after removing this sample (result not shown), there was no significant improvement in the model's performance.

Despite this, the analysis of other diagnostic measures (e.g., leverage, studentized residuals, Hotelling's T^2) did not identify any outliers, leading to the conclusion that the non-normality in the jet fuel residuals does not significantly undermine the model's reliability or the validity of the predictions.

Despite the higher-than-expected relative errors in some cases, particularly in samples with low TAN values, the overall model performance remained robust. The retained samples, including those with high relative errors, did not unduly distort the model, and the normal distribution of residuals further validates the reliability of the predictions. This thorough analysis ensures that the models can be confidently used for predicting TAN values across a wide range of crude oil samples and distillation cuts.

While the thorough evaluation of potential outliers was a critical aspect of validating the model, it is essential to recognize that the elevated absolute relative errors observed in some samples, particularly those with low TAN values, were not indicative of data anomalies. Instead, these higher errors can be a direct consequence of the TAN values being close to the quantification limit of the reference method used[2]. This proximity to the method's quantification threshold naturally increases the relative error, without necessarily reflecting true deviations or anomalies in the data. It underscores the inherent limitations of the reference method at lower TAN values, where precision and reliability are inherently compromised. By acknowledging this factor, we avoided the erroneous exclusion of valuable data, thereby maintaining the integrity and representativeness of the dataset. This consideration is crucial, as it highlights the importance of a meticulous analysis of measurement errors and the interpretation of results, particularly when working with data near critical limits defined by reference methods. Consequently, the robustness of the models is affirmed, and the findings underscore the necessity of accounting for the technical limitations of measurement methods in the analysis of analytical data.

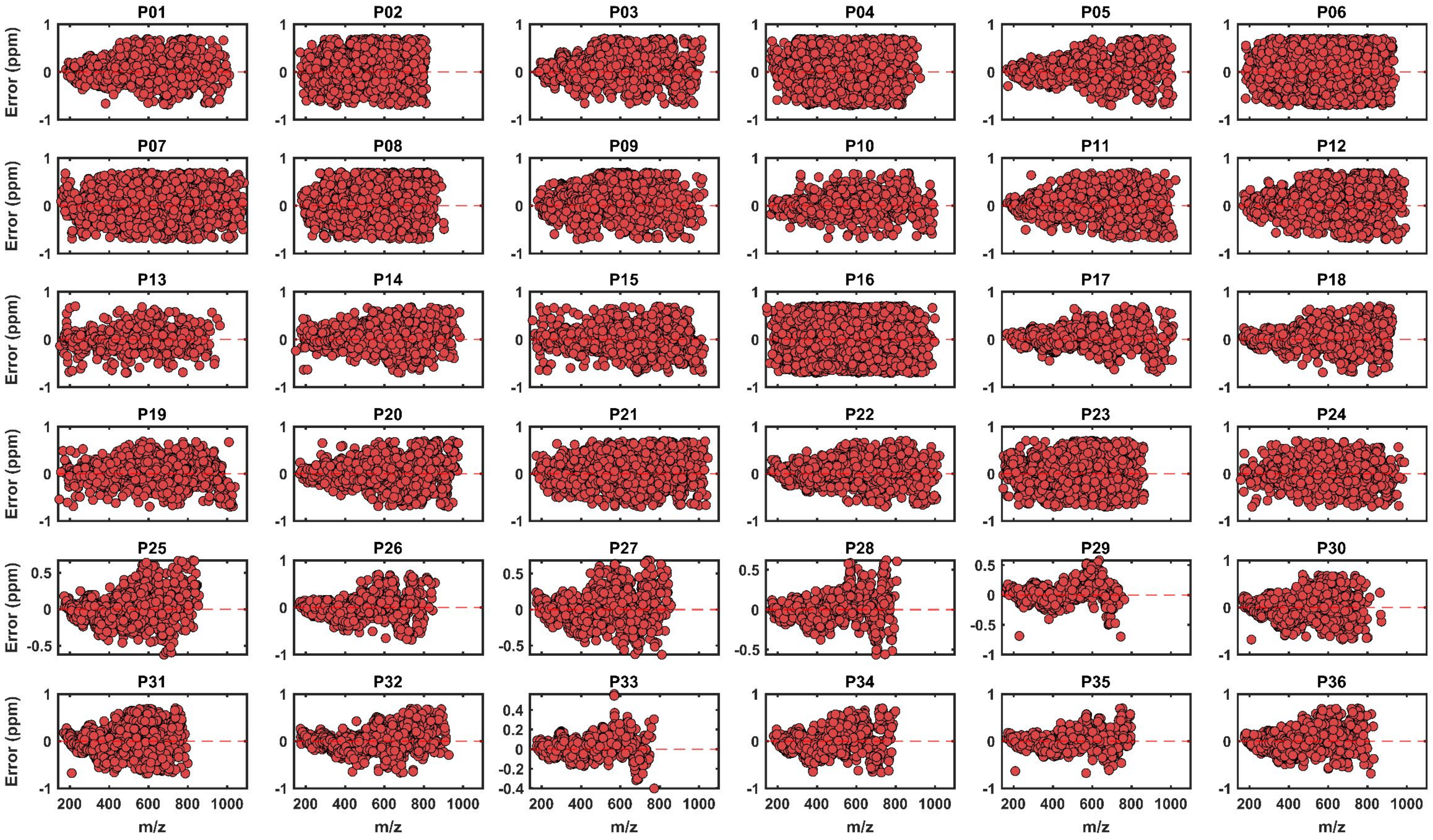

Figure S1. Error distribution as a function of *m/z* for the 36 crude oil samples analyzed by ESI (−) FT-ICR MS.

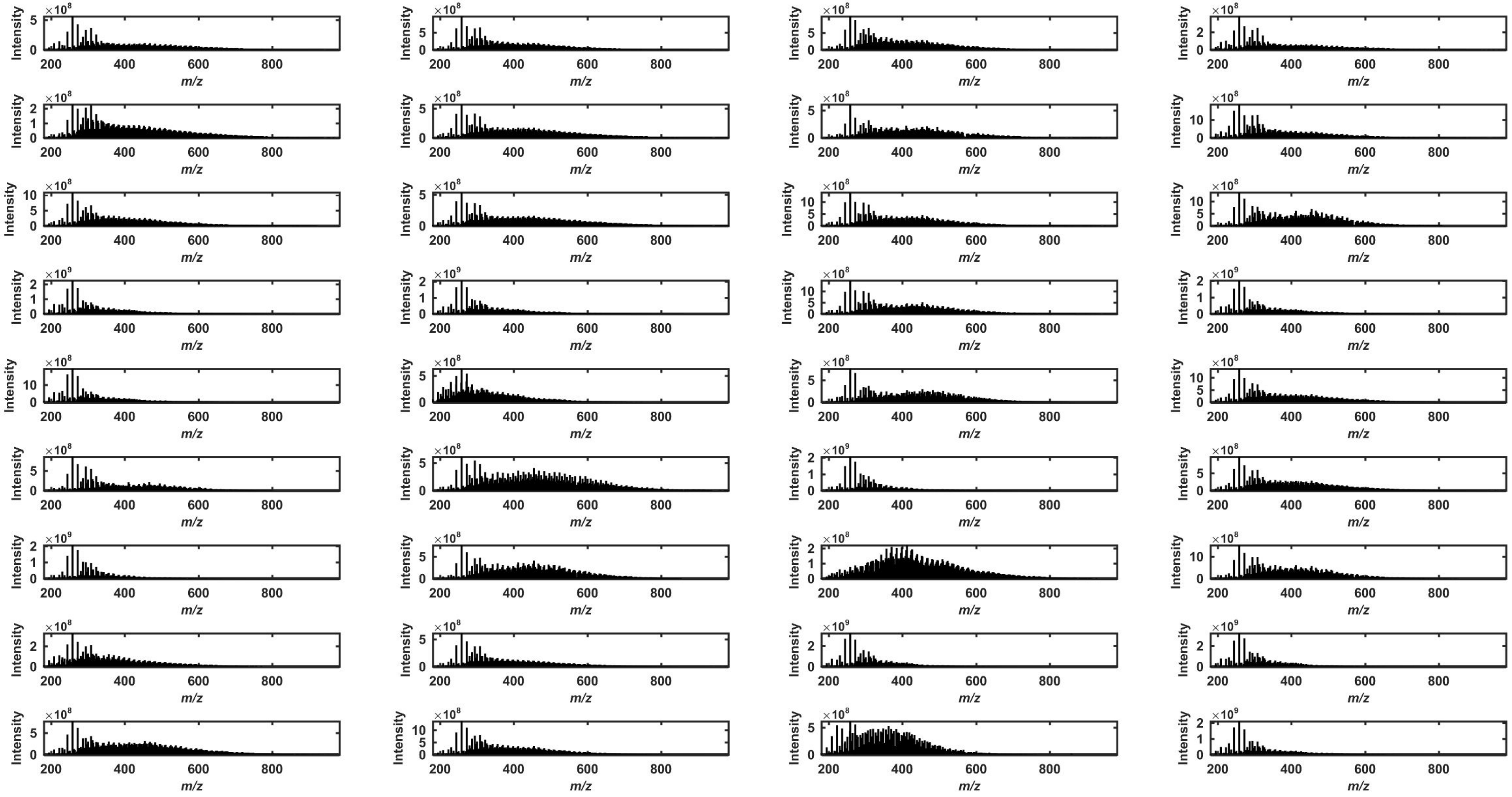

Figure S2. ESI (−) FT-ICR MS spectra of 36 crude oil samples. Each subplot represents the mass spectrum of an individual sample, displaying the intensity of ion signals (y-axis) as a function of their mass-to-charge ratio (*m/z*) (x-axis). The spectra provide a comprehensive overview of the chemical composition of each crude oil sample, highlighting the diversity and complexity within the dataset.

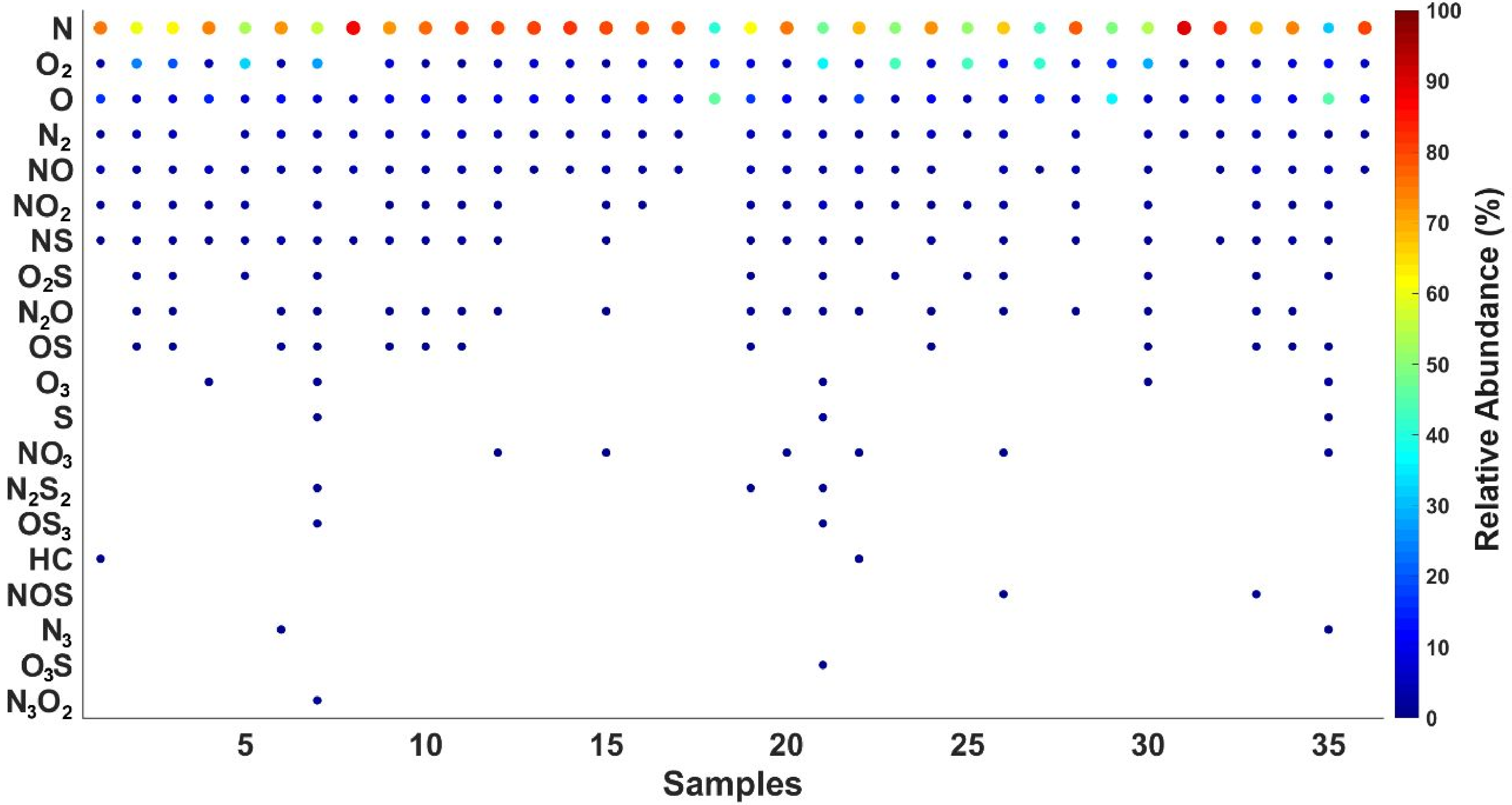

Figure S3. Class distribution of 20 compound classes in the crude oil samples. The *y*-axis lists the various compound classes, including hydrocarbons (HC), nitrogen-containing compounds (N), and various combinations of nitrogen, oxygen, and sulfur-containing compounds (e.g.,

N_2, N_2O, N_2S, O_2, O_2S). The *x*-axis shows the samples. The color bar indicates the relative abundance, with red representing the highest abundance and blue representing the lowest. This figure highlights the variability and complexity of the chemical composition within the crude oil samples.

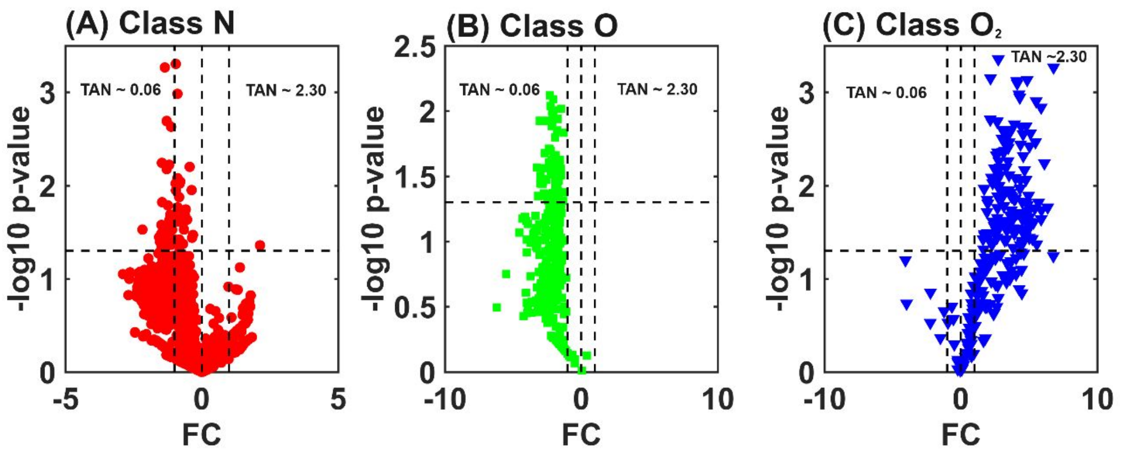

Figure S4. Volcano plots comparing the low (0.06 mg KOH g^{-1} oil) and high (2.3 mg KOH g^{-1} oil) TAN values in crude oil samples for the three most prominent classes: (A) class N, (B) class O, and (C) class O_2. The *x*-axis represents the fold change (FC) between the two TAN groups, while the *y*-axis shows the negative logarithm of the p-value (-log10 *p*-value).

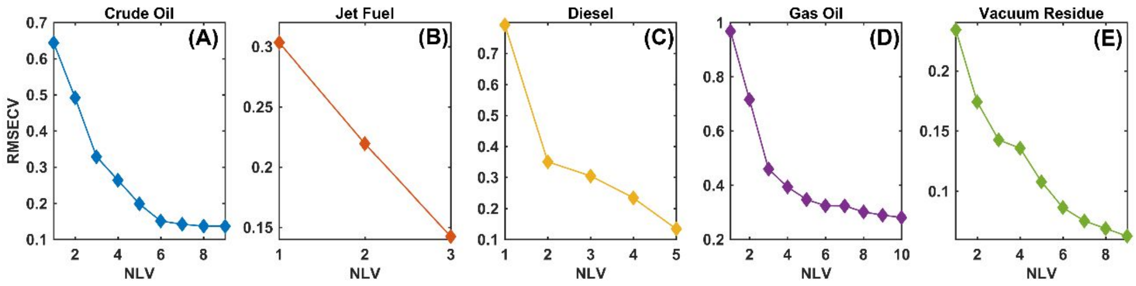

Figure S5. RMSECV as a function of the number of latent variables (NLV) for crude oil and its distillation cuts. (A) Crude Oil, (B) Jet Fuel, (C) Diesel, (D) Gas Oil, and (E) Vacuum Residue. The plots demonstrate the optimization process for selecting the optimal NLV for each model using 10-fold cross-validation within the calibration set. The RMSECV values decrease with increasing NLV, reflecting improved model performance until an optimal balance is achieved.

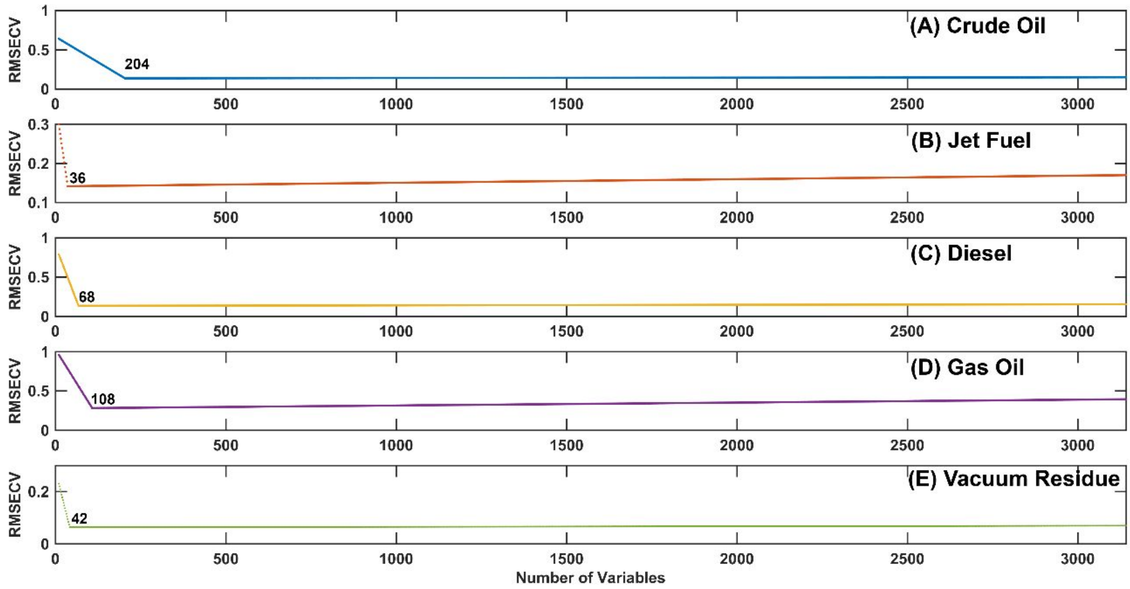

Figure S6. Evolution of RMSECV as a function of the number of variables selected by OPS for crude oil and its distillation cuts. (A) Crude Oil, (B) Jet Fuel, (C) Diesel, (D) Gas Oil, and (E) Vacuum Residue. The plots illustrate the relationship between the number of selected variables and the RMSECV during the variable selection process. The initial sharp drop in RMSECV demonstrates the effectiveness of OPS in identifying the most relevant variables for model building.

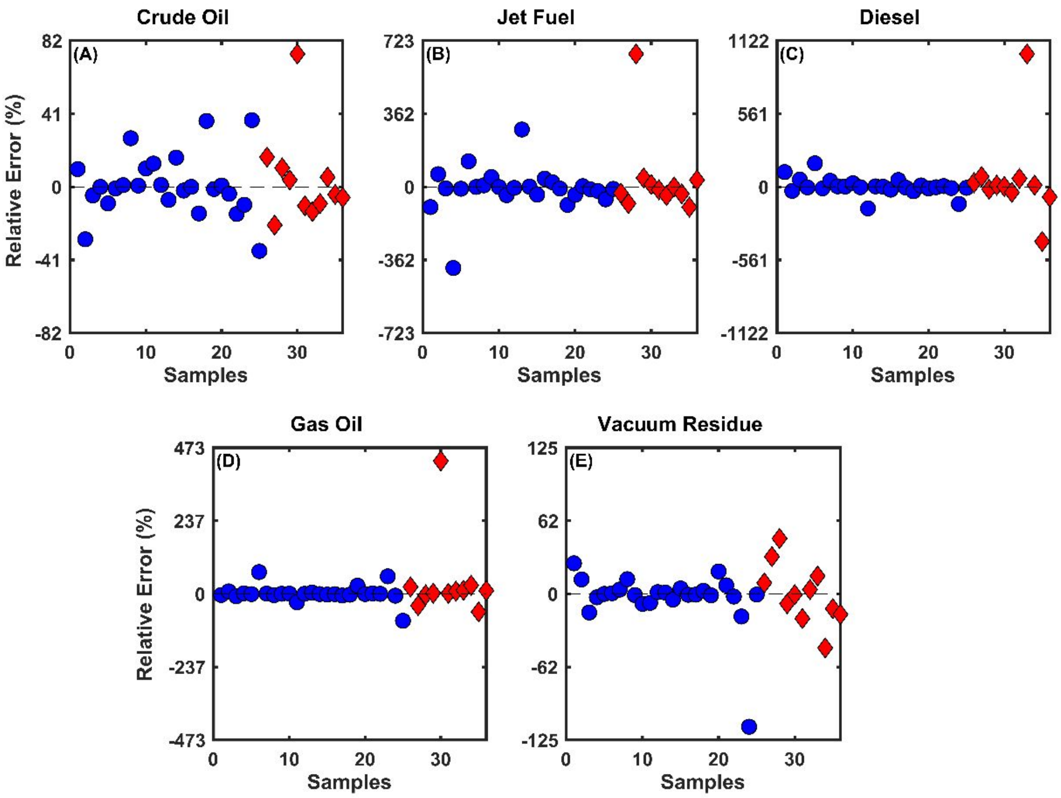

Figure S7. Relative error plots for crude oil and its distillation cuts. (A) Crude Oil, (B) Jet Fuel, (C) Diesel, (D) Gas Oil, and (E) Vacuum Residue. The relative errors (%) are plotted for each sample, with blue circles representing calibration set samples and red diamonds representing validation set samples. These plots illustrate the distribution of relative errors across the dataset, highlighting the accuracy of the predictive models and any discrepancies between the calibration and validation sets.

The relative errors are calculated as the ratio of the absolute difference between the measured reference and the predicted TAN values to the measured reference value, typically expressed as a percentage. These relative errors provide an indication of the prediction accuracy for each sample. A relative error value close to zero suggests high accuracy in the prediction, while larger relative error values indicate a greater discrepancy between the predicted and measured values.

The analysis of relative errors revealed that higher errors were predominantly observed for samples with low TAN values. This behavior can be attributed to the proximity of these samples to the quantification limit of reference method, where variability is inherently greater. Despite these higher relative errors, the model demonstrated robust performance across the critical range of TAN values, particularly for samples with moderate to high TAN levels, which are of greater significance in industrial applications.

For refining processes, accurate prediction of higher TAN values is crucial, as these samples are more likely to contribute to operational challenges such as corrosion and require specific mitigation strategies. The ability of the model to reliably predict TAN values in this critical range underscores its practical applicability.

While the accuracy for low TAN samples could be improved with further refinements or additional replicates, the current model provides sufficient reliability for its intended application. This highlights the balance between predictive accuracy and practical utility, with the model effectively addressing the needs of industrial operations while acknowledging areas for future improvement.

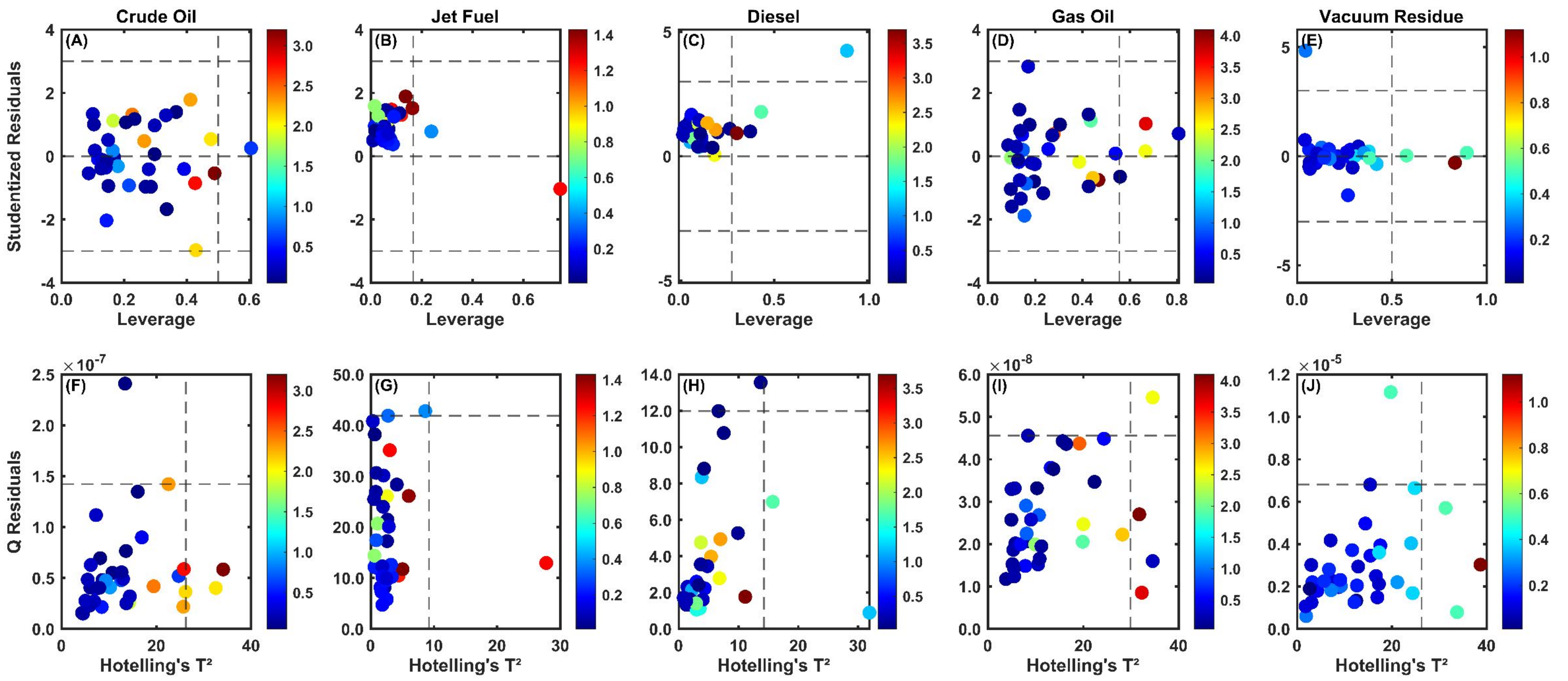

Figure S8. Diagnostic plots for outlier detection in the predictive models for crude oil and its distillation cuts. The top row (A-E) shows leverage versus studentized residuals plots for (A) Crude Oil, (B) Jet Fuel, (C) Diesel, (D) Gas Oil, and (E) Vacuum Residue. Leverage indicates the influence of each observation on the model, while studentized residuals assess the accuracy of the model's predictions. Observations outside the dashed lines represent potential outliers. The bottom row (F-J) presents Hotelling's T^2 versus Q residuals plots for the same samples, where Hotelling's T^2 measures the multivariate distance of observations in the predictor space, and Q residuals indicate how well the observations fit the model. Points exceeding the threshold lines suggest deviations that may impact the model. The color scale represents the Total Acid Number (TAN) values for the respective samples, providing additional context for the identified patterns. These plots collectively aid in identifying potential outliers and assessing the robustness of the predictive models.

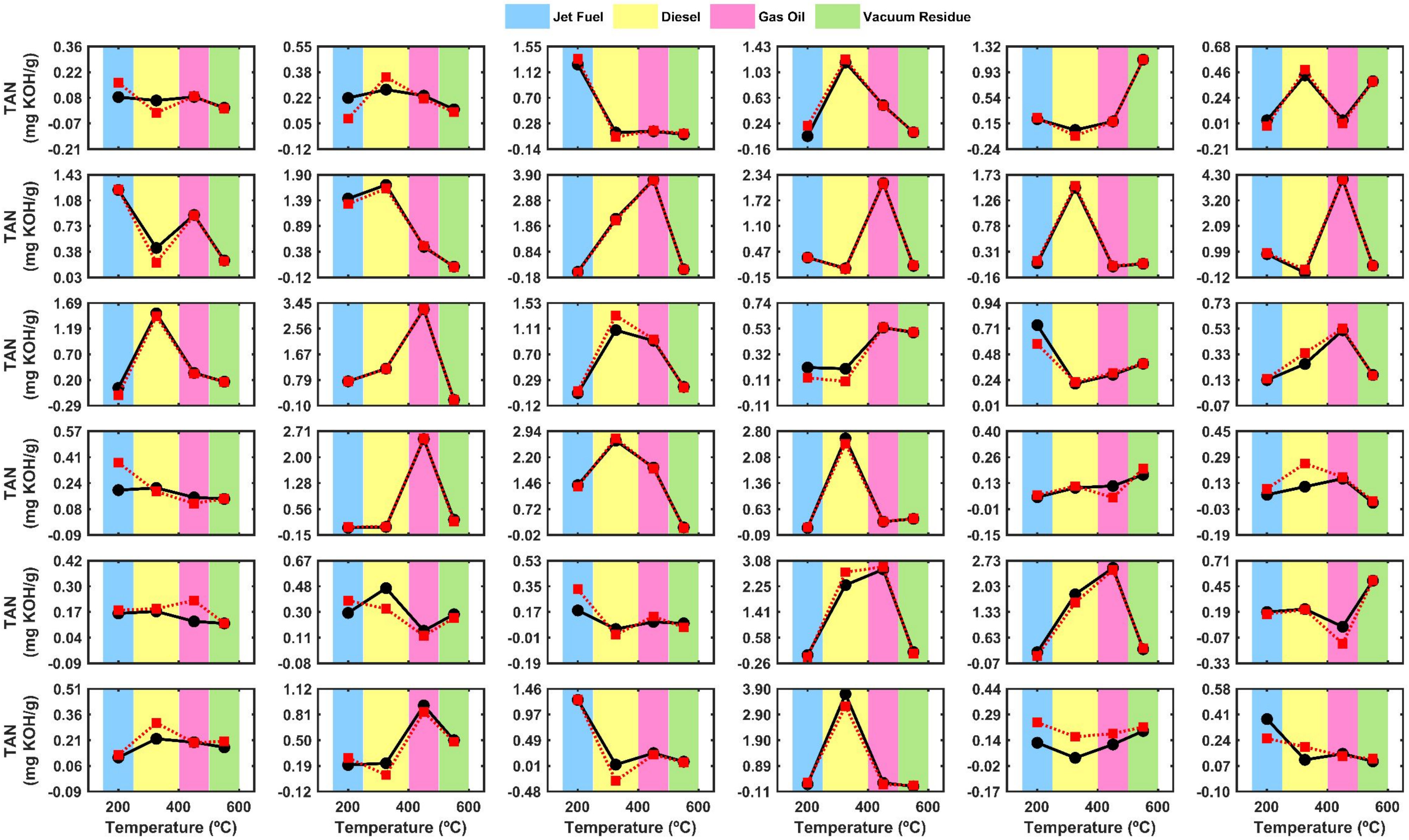

Figure S9. TAN boiling point (BP) distribution for all 36 crude oil samples. The *x*-axis represents the approximate average temperature range corresponding to the boiling points of the cuts: jet fuel (150 – 250 ºC), diesel (250 – 400 ºC), gas oil (400 – 500 ºC), and vacuum residue (500 – 600

ºC). The black solid line represents the reference TAN values measured by titration, while the red dashed line shows the TAN values predicted by the model.

References

(1) Zeng, Y.; Chen, X.; Zhao, D.; Li, H.; Zhang, Y.; Xiao, X. Estimation of PKa Values for Carboxylic Acids, Alcohols, Phenols and Amines Using Changes in the Relative Gibbs Free Energy. *Fluid Phase Equilib.* **2012**, *313*, 148–155. https://doi.org/10.1016/j.fluid.2011.09.022.

(2) ASTM D664-09. Standard Test Method for Acid Number of Petroleum Products by Potentiometric Titration. ASTM International: West Conshohocken, PA 2006. https://doi.org/10.1520/D0664-18E02.
